# Role of Cardiovascular Imaging in Risk Assessment: Recent Advances, Gaps in Evidence, and Future Directions

**DOI:** 10.3390/jcm12175563

**Published:** 2023-08-26

**Authors:** Francesco Perone, Marco Bernardi, Alban Redheuil, Dario Mafrica, Edoardo Conte, Luigi Spadafora, Fiona Ecarnot, Lale Tokgozoglu, Carlos G. Santos-Gallego, Sergio Emanuel Kaiser, Federica Fogacci, Annabelle Sabouret, Deepak L. Bhatt, Francesco Paneni, Maciej Banach, Raul Santos, Giuseppe Biondi Zoccai, Kausik K. Ray, Pierre Sabouret

**Affiliations:** 1 Cardiac Rehabilitation Unit, Rehabilitation Clinic “Villa delle Magnolie”, Castel Morrone, 81020 Caserta, Italy; francescoperone1988@gmail.com; 2 Department of Clinical, Internal Medicine, Anesthesiology and Cardiovascular Sciences, Sapienza University of Rome, 00185 Rome, Italy; marco.bernardi23@gmail.com (M.B.); dariomafrica@gmail.com (D.M.); luigispadafora167@gmail.com (L.S.); 3 Laboratoire d’Imagerie Biomédicale, Sorbonne University, INSERM 1146, CNRS 7371, 75005 Paris, France; alban.redheuil@aphp.fr; 4 Cardiology Department, Galeazzi-Sant’Ambrogio Hospital IRCCS, 20100 Milan, Italy; edoardo.conte86@gmail.com; 5 Department of Cardiology, University Hospital Besancon, University of Franche-Comté, 25000 Besancon, France; fiona.ecarnot@univ-fcomte.fr; 6 Department of Cardiology, Medical Faculty, Hacettepe University, 06230 Ankara, Turkey; laletok@gmail.com; 7 Atherothrombosis Research Unit, Icahn School of Medicine at Mount Sinai, New York, NY 10029, USA; carlosgsantos.gallego@gmail.com; 8 Mount Sinai Heart, Icahn School of Medicine at Mount Sinai Health System, New York, NY 10029, USA; dlbhattmd@post.harvard.edu; 9 Discipline of Clinical and Experimental Pathophysiology, Rio de Janeiro State University, Rio de Janeiro 23070-200, Brazil; kaiser.trp@terra.com.br; 10 Hypertension and Cardiovascular Risk Research Group, Medical and Surgical Sciences Department, Alma Mater Studiorum University of Bologna, 40126 Bologna, Italy; federicafogacci@gmail.com; 11 Gustave-Roussy Institute, Hérault Department, 94805 Villejuif, France; annabelle.sabouret@gmail.com; 12 Department of Cardiology, University Heart Center, University Hospital Zurich, 8091 Zurich, Switzerland; francesco.paneni@usz.ch; 13 Center for Translational and Experimental Cardiology (CTEC), University Hospital Zurich and University of Zurich, 8091 Zurich, Switzerland; 14 Department of Preventive Cardiology and Lipidology, Medical University of Lodz (MUL), Rzgowska 281/289, 93-338 Lodz, Poland; maciejbanach77@gmail.com; 15 Cardiovascular Research Centre, University of Zielona Gora, 65-417 Zielona Gora, Poland; 16 Heart Institute, University of Sao Paulo Medical School, São Paulo 05403-903, Brazil; raul.santos@incor.usp.br; 17 Department of Medical-Surgical Sciences and Biotechnologies, Sapienza University of Rome, 00185 Roma, Italy; gbiondizoccai@gmail.com; 18 Mediterranea Cardiocentro, 80122 Napoli, Italy; 19 Imperial Centre for Cardiovascular Disease Prevention and Imperial Clinical Trials Unit, Department of Public Health and Primary Care, Imperial College London, London SW7 2BX, UK; koshray@gmail.com; 20 Heart Institute, Cardiology Department, Paris and National College of French Cardiologists, Pitié-Salpétrière Hospital, Sorbonne University, 75013 Paris, France

**Keywords:** cardiovascular imaging, risk assessment, prevention, cardiovascular risk

## Abstract

Optimal risk assessment for primary prevention remains highly challenging. Recent registries have highlighted major discrepancies between guidelines and daily practice. Although guidelines have improved over time and provide updated risk scores, they still fail to identify a significant proportion of at-risk individuals, who then miss out on effective prevention measures until their initial ischemic events. Cardiovascular imaging is progressively assuming an increasingly pivotal role, playing a crucial part in enhancing the meticulous categorization of individuals according to their risk profiles, thus enabling the customization of precise therapeutic strategies for patients with increased cardiovascular risks. For the most part, the current approach to patients with atherosclerotic cardiovascular disease (ASCVD) is homogeneous. However, data from registries (e.g., REACH, CORONOR) and randomized clinical trials (e.g., COMPASS, FOURIER, and ODYSSEY outcomes) highlight heterogeneity in the risks of recurrent ischemic events, which are especially higher in patients with poly-vascular disease and/or multivessel coronary disease. This indicates the need for a more individualized strategy and further research to improve definitions of individual residual risk, with a view of intensifying treatments in the subgroups with very high residual risk. In this narrative review, we discuss advances in cardiovascular imaging, its current place in the guidelines, the gaps in evidence, and perspectives for primary and secondary prevention to improve risk assessment and therapeutic strategies using cardiovascular imaging.

## 1. Introduction

Despite major progress in recent decades, cardiovascular diseases (CVDs) still represent the leading cause of mortality and morbidity worldwide [1]. Indeed, after an initial dramatic fall in cardiovascular (CV) deaths in the early phase of acute coronary syndromes (ACSs), the decline in mortality is slowing, while CVD morbidity is rising due to the increasing prevalence of risk factors [2]. These findings indicate that better identification of individuals with high risk for primary prevention is necessary to facilitate earlier action and reduce the burden of major adverse cardiovascular events (MACE). The leading modifiable risk factors are smoking, type 2 diabetes, dyslipidemia, and high blood pressure, which are often associated with poor lifestyle in terms of diet and exercise, culminating in a high prevalence of metabolic syndrome and obesity worldwide [3].

Screening for CV disease is crucial since atherosclerosis is a long, progressive, and silent process that occurs prior to the index acute event in most individuals. Since the first clinical manifestation of CVD is acute myocardial infarction (AMI) or sudden cardiac death in 50% of individuals, early identification of high-risk individuals is a major issue in the implementation of effective prevention strategies in the community [4].

The European Society of Cardiology (ESC) guidelines have recently been updated, and they recommend the use of the SCORE2 and SCORE2-OP risk assessment tools [5,6]. The American College of Cardiology (ACC) guidelines have updated the atherosclerotic cardiovascular disease (ASCVD) risk assessment score [7] with the common goal of enabling enhanced risk assessment of the European and US populations. As mentioned, SCORE2 is a new algorithm that was derived, calibrated, and validated for the prediction of the 10-year risk of first-onset CVD in European populations. SCORE2-OP (older populations) was developed in a similar manner, with the aim of predicting the 10-year risk of a CVD event in people over 65 years of age. However, as risk factor scores are based on probabilistic calculations derived from population-based studies, these improved risk scores do not apply to all individuals. Furthermore, these risk assessment tools have some weaknesses, which may explain why recent data show that they fail to identify some high-risk individuals [8]. These scores represent an undisputed step forward in risk evaluation but remain suboptimal since they fail to yield accurate evaluations in several subgroups of patients, including those with a family history of premature CAD, individuals with chronic inflammatory diseases, and users of recreational drugs. Additionally, evaluation of the duration of exposure to risk factors is also suboptimal.

Concerning non-modifiable risk factors, scores tend to underestimate risk in younger individuals and women. Emerging non-traditional risk factors and/or markers have been mentioned, but strategies for detection, risk evaluation, and management are not well defined, underscoring the need for further research in this field. These markers include apolipoprotein A (ApoA), apolipoprotein B (ApoB), high-sensitivity C-reactive protein (hs-CRP), brain natriuretic peptides (BNP), troponin I homocysteine, interleukins 1 and 6 (IL1, IL6), lipoprotein (a) [Lp(a)], cholesterol remnants, size and number of low-density lipoprotein (LDL) particles, tissue/tumor necrosis factor-α (TNF- α), and uric acid [9]. All these factors and markers are associated with an increased risk of MACE, but their precise place in the risk assessment and the optimal strategy for addressing them requires better definition.

Since the ASCVD substrates are plaque characteristics and progression, monitoring the progression of atheroma using CV imaging techniques seems to be a promising and logical approach to ensure patients are treated adequately. Progress in the development of cardiovascular imaging supports its growing role as a modern strategy for improving risk classification and optimizing therapeutic strategies for high-risk patients. However, the role of CV imaging in risk assessment for primary and secondary prevention is not well established in the current guidelines.

In this narrative review, we propose a critical discussion and overview of risk assessment for primary and secondary prevention using CV imaging. Specifically, we highlight the advances in cardiovascular imaging to improve risk assessment, the current place of imaging in the guidelines, the gaps in evidence, as well as future directions.

## 2. Cardiovascular Imaging for Primary Prevention

For years, CVD prevention has been the focus of the medical community. Primary prevention plays a key role in reducing the incidence of CVDs in the population. Due to continuous progress and better access, CV imaging should play a key role in identifying individuals with high risks of CVDs beyond the traditional approach based on risk scores [10]. Both European and American scientific associations have published recommendations on cardiovascular imaging for primary prevention [7,11,12]. The guidelines recommend that non-invasive imaging techniques, such as contrast computed tomography (CT) coronary angiography, coronary artery calcium (CAC) score, and carotid ultrasound (US), should be considered during risk assessment for primary prevention. Technology advancements have helped reduce the amount of radiation exposure, and all of these techniques ensure the highest level of safety [13]. Current guidelines emphasize the importance of using cardiovascular imaging alongside traditional risk factor assessment, not in place of it. Furthermore, the guidelines caution that imaging should be tailored to individual patient characteristics [12]. CV risk can be assessed using non-invasive imaging strategies, including echocardiography, CAC scores, CT scans, magnetic resonance imaging (MRI), and nuclear imaging. It is possible to detect subclinical disease, monitor disease progression, or rule out coronary artery disease (CAD) using these techniques, but guidelines do not provide specific guidance on the management of individuals for primary prevention.

### 2.1. Coronary Computed Tomography Angiography (CCTA)

**Figure 1:**
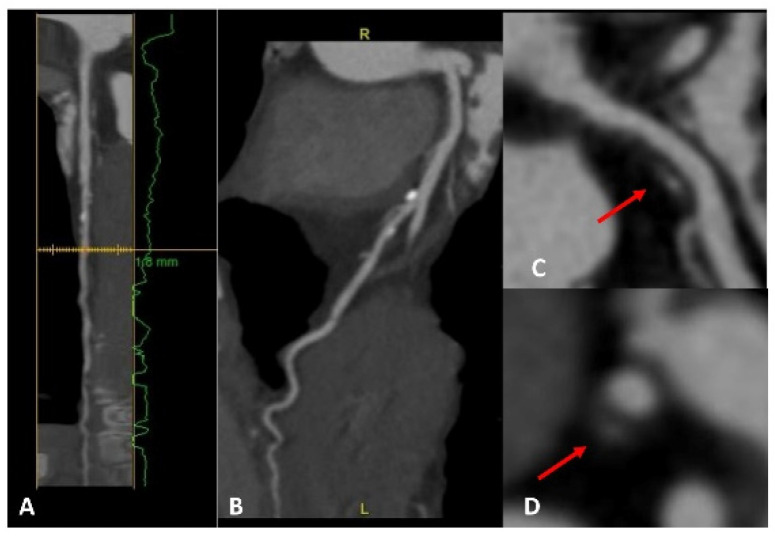
A patient with a strong family history of CAD, with cardiac CT positive for non-obstructive CAD on proximal LAD (panels **A**,**B**). A high-risk fibrolipidic plaque (red arrow) with positive remodeling, low attenuation, and small spotty calcification is evident in both the long (panel **C**) and short (panel **D**) axis views.

Coronary computed tomography angiography (CCTA) is a non-invasive imaging test widely used in daily practice. Its validity and feasibility in the diagnosis and risk stratification of CAD are well established (Figure 1). CCTA has replaced coronary angiography as a diagnostic tool for confirming CAD. Indeed, CCTA is useful for ruling out CAD with an intermediate or low pre-test probability due to its high negative predictive value (NPV) [14]. Numerous randomized controlled trials (RCTs) have confirmed the effectiveness and potency of CCTA in this field [15,16,17].

### 2.2. Coronary Artery Calcium Score (CAC Score)

Currently, one of the most validated tools for managing primary prevention in asymptomatic patients is the CAC score derived from CT scans [18]. The CAC score is an estimate of the amount of calcium within the walls of the coronary arteries as determined using a non-invasive imaging procedure (CCTA). CAC reliably indicates CAD and cardiovascular events in asymptomatic individuals [19,20]. Indeed, coronary calcification and CVDs are strongly correlated, and both ESC and ACC/AHA guidelines currently approve the use of CAC scoring. The CAC score is commonly used to assess risk stratification, and a CAC = 0 is a strong marker of low risk [7,12]. Numerous studies have evaluated the use of CAC scores for primary prevention:

In the Prospective Army Coronary Calcium Project conducted in 2005, CAC showed an incremental predictive value for premature CVD outcomes in a cohort of healthy men and women [21].

The Multi-Ethnic Study of Atherosclerosis (MESA) confirmed the efficacy of the CAC score as a strong predictor of coronary heart disease, irrespective of ethnicity [22].

The validity of the CAC score was also evaluated in the Framingham population, where it appeared to be associated with CVD, independent of Framingham risk factors [23].

Additional evidence was reported in a statement from the CAC Consortium, where the CAC score was shown to be the most reliable predictor of long-term mortality [24].

Based on the 2021 ESC guidelines, CAC scoring may be considered to improve risk classification (class IIb) [11]. If CAC scoring is not available, it can be replaced by carotid ultrasound, even though CAC better predicts cardiovascular events [25].

The 2019 ESC guidelines on chronic coronary syndromes recommend the use of CAC in patients with intermediate-risk factors for CAD who have doubtful or inconclusive stress testing or who have symptoms suggestive of CAD but normal stress testing results [26]. The guidelines suggest that a CAC score = 0 can effectively exclude the presence of significant CAD, whereas a CAC score > 400 indicates a high probability of significant CAD. The ACC/AHA also recommends the use of the CAC score as a diagnostic tool for assessing the presence and severity of CAD. In line with the ESC guidelines, the ACC/AHA recommendations suggest the use of CAC in patients with intermediate-risk factors for CAD [7]. Nevertheless, there are some differences between the two sets of guidelines. The ESC guidelines recommend using CAC scoring as an additional tool for risk assessment in asymptomatic individuals with intermediate cardiovascular risk [12]. The ACC/AHA guidelines also recommend CAC scoring, but only in individuals with intermediate risk who are undecided about statin therapy after clinician–patient risk discussion [7]. In addition, the ESC guidelines use a CAC score of 100 Agatston units or more as the threshold for identifying individuals who may benefit from statin therapy. In contrast, the ACC/AHA guidelines use a threshold of the 75th percentile or greater for age, sex, and ethnicity as the threshold for considering statin therapy. The ACC/AHA guidelines provide specific CAC scoring thresholds for African American, Hispanic/Latino, and South Asian individuals, while the ESC guidelines do not differentiate by ethnicity [7,12]. The ESC guidelines advise against using CAC scoring in low-risk individuals, while the ACC/AHA guidelines do not recommend routine CAC scoring in low-risk individuals but acknowledge that some individuals may choose to have the test performed for personal reasons [7,12].

Overall, both the ESC and ACC/AHA guidelines support the use of CAC scoring as a risk assessment tool for primary prevention. Clinicians should consider the guidelines in conjunction with their clinical judgment and individual patient factors when deciding whether to use CAC scoring as part of primary prevention efforts. It has also been shown that CAC scoring can be useful in patients with familial hypercholesterolemia (FH), a genetic disorder characterized by high LDL cholesterol levels. A study published in 2019 found that CAC scoring was effective at identifying FH patients at high risk of cardiovascular events and could guide treatment decisions in this population [27]. Nevertheless, while some studies have suggested that CAC scoring can be useful in certain patient populations, the ESC guidelines do not currently recommend the routine use of CAC scoring in patients with FH [12]. Thus, additional research is needed to determine the most effective ways of using CAC scoring to assess cardiovascular risk in different patient populations.

Despite its feasibility and cost-effectiveness, CAC has some limitations that need to be addressed in future studies. Indeed, the use of CAC is still debated in younger adults, particularly those under the age of 40, in whom coronary artery calcification is less common [28]. This means that CAC scoring may not be as useful for risk assessment in these individuals. Additionally, it is important to consider the harmful effects of ionizing radiation exposure in younger individuals who are more sensitive to these effects [29]. Another crucial aspect to consider is that CAC scoring is not widely available in all healthcare settings and may not be covered by all insurance plans [30]. Undoubtedly, there is a pressing need for a novel CAC score encompassing a comprehensive range of factors, including age-specific percentiles, tissue density, surface area, anatomical locations, vessel count, and extra-coronary calcifications. Currently, the majority of CAC scoring systems are rooted in the Agatston score, which solely assesses the quantity of calcium present in the coronary arteries. The integration of these proposed items introduces complexities in terms of data acquisition, processing, and interpretation. Furthermore, variabilities in imaging techniques, patient characteristics, and data sources require a rigorous and concerted effort to standardize methodologies and establish robust correlations with clinical outcomes. Nevertheless, we consider this strategy of implementing the currently available scores pivotal.

### 2.3. Other Imaging Techniques

Other imaging techniques are considered in ESC guidelines for primary prevention of CV to improve risk stratification. Atherosclerosis is a progressive disease that can involve multiple vessels and areas of the body and includes peripheral artery disease (PAD). PAD can affect the extremities, abdominal aorta, and carotid arteries; this latter localization may lead to stroke. To stage CV disease, imaging techniques such as US imaging can help assess the presence and extent of atherosclerotic plaques at the carotid, abdominal, and peripheral levels. Regarding carotid artery imaging, measurements of carotid plaques have demonstrated the best prognostic value. Through the utilization of imaging to identify and monitor the progression of CVDs, it is possible to individualize risk assessment for each patient at an early stage. Furthermore, through ultrasound (US) imaging, it is possible to document atherosclerosis in terms of the composition of the plaque (fat or calcium predominance), the plaque burden, and the potential hemodynamic influence. Moreover, imaging enables earlier detection of CVD in younger patients whose risk is often underestimated by scores. Carotid ultrasound is recommended (class IIb) as a risk modifier when a CAC score is not applicable [31]. The measurement of carotid artery intima-media thickness (IMT) is not recommended as a risk modifier in the guidelines due to the lack of a standardized measurement and cut-off and represents more arterial remodeling than atherosclerosis. There are some lines of evidence suggesting that carotid IMT is correlated with CV events [32]. Therefore, carotid ultrasound is the preferred method for predicting atherosclerosis progression [33].

The ankle–brachial index (ABI) is a simple, non-invasive, and cheap test that measures the ratio of systolic blood pressure in the ankle to that in the arm. The ABI is a useful tool for risk assessment in individuals at intermediate or high risk of cardiovascular disease. The guidelines recommend measuring the ABI in individuals with suspected or established PAD, as well as in those with symptoms of claudication (leg pain when walking) or atypical leg symptoms. In a study involving 5003 older adults, it was shown that participants with no history of ASCVD had a greater risk of CHD and stroke when the ABI was ≤ 0.9 [34]. However, the ABI is not recommended as a routine screening tool in asymptomatic individuals as there is limited evidence of its effectiveness in primary prevention [11].

Echocardiography is also not recommended as a routine screening tool for primary prevention of cardiovascular disease in asymptomatic individuals [11]. However, it may be considered part of a comprehensive CV risk assessment in individuals with suspected or established cardiovascular disease.

In specific cohorts, such as athletes or individuals at elevated risk of sudden cardiac death, echocardiography assumes a paramount role in primary cardiovascular prevention, facilitating targeted assessment and early detection of potential cardiac issues, ultimately contributing to informed decision-making and personalized care [35,36].

**Figure 2:**
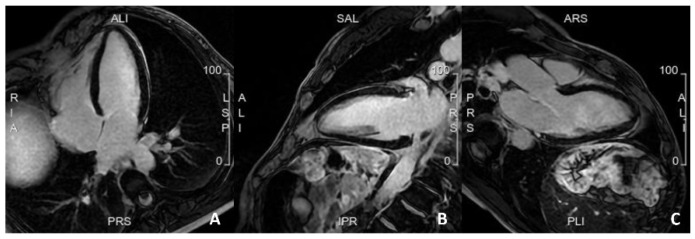
A case of a patient with a family history of sudden cardiac death, with frequent but isolated ectopic ventricular beats. Cardiac MRI was completely normal, with no evidence of myocardial fibrosis. (**A**). 4 Chamber view; (**B**). 2 Chamber view; (**C**). 3 Chamber view.

Another imaging technique that may play a prominent role in the future is cardiac magnetic resonance imaging (CMR). Indeed, CMR imaging has become an essential tool in diagnosing and managing cardiovascular diseases. It provides precise information on the structure and function of the heart, including the size and thickness of the heart walls, stroke volume, as well as any abnormalities in the heart muscle [37]. Thus, CMR can be used to detect early signs of myocardial damage or fibrosis, which may indicate an increased risk of heart failure or sudden cardiac death [38] (Figure 2). Consequently, CMR can be used to guide the implantation of an Implantable Cardioverter Defibrillator (ICD) for primary prevention [39]. Additionally, CMR can be used to assess valve function and identify structural abnormalities associated with a higher risk of cardiovascular events [40]. Overall, CMR has the potential to play an important role in primary prevention by providing more accurate risk stratification and guiding personalized management strategies for individuals at increased risk of cardiovascular disease. However, there is a lack of evidence on this topic, and the use of CMR to guide ICD implantation for primary prevention needs to be tested in RCTs. Notably, the ‘Cardiac Magnetic Resonance Guidance of Implantable Cardioverter Defibrillator Implantation in Non-ischemic Dilated Cardiomyopathy (CMR-ICD)’ trial is slated to complete recruitment by November 2023. This study is poised to shed light on the potential benefits of CMR for primary prevention to guide the implantation of ICD. The rationale is that CMR allows a meticulous assessment of cardiac functionality, morphology, and tissue characteristics [41]. The prospect of leveraging CMR, including the evaluation of late gadolinium enhancement (LGE), holds promising implications for enhancing risk stratification and decision-making in this cohort. The feasibility and cost-effectiveness of CMR compared with other imaging modalities also require evaluation.

## 3. Risk Assessment Using Cardiovascular Imaging for Secondary Prevention

CV risk assessment in patients with ASCVD is needed to estimate the risk of further CV events. For secondary prevention, this is termed “residual” risk, and patients are at high risk of recurrent events. Reducing the global CV disease burden and risk of new events requires personalized and intensified treatment [11]. Specifically, residual risk in these patients is calculated using risk stratification tools to define the 10-year risk of recurrent CV disease. Currently, the SMART (Secondary Manifestations of Arterial Disease) risk score is recommended for estimating risk for secondary prevention. This score is calculated in patients with CAD, PAD, abdominal aortic aneurysm (AAA), or cerebrovascular disease [42]. The variables needed to calculate the 10-year risk are age, sex, smoking status, diabetes mellitus, systolic blood pressure, total and high-density lipoprotein cholesterol, creatinine, high-sensitivity C-reactive protein, years since the first CV event, and form of the disease (CAD, PAD, AAA, or cerebrovascular disease).

The European guidelines for cardiovascular prevention consider patients with ASCVD to be at high or very high risk [11]. Residual CV risk assessment is not based on information and parameters from CV imaging; this is a notable gap in risk assessment. CV imaging can stratify patients for secondary prevention, rather than considering them all as constituting a single category. Furthermore, careful stratification should also be conducted for poly-vascular patients. Generally, poly-vascular status is associated with excess MACE despite medical treatment, and, in these cases especially, imaging could add further relevant prognostic stratification. For example, CAD plus 50% carotid stenosis is associated with a 100% increase in MACE, as suggested by the REACH registry [43,44].

CV imaging, such as echocardiography, coronary CT, and CMR, could be key exams for stratifying patients after CV events, ultimately improving therapeutic strategies and preventing further events.

**Figure 3:**
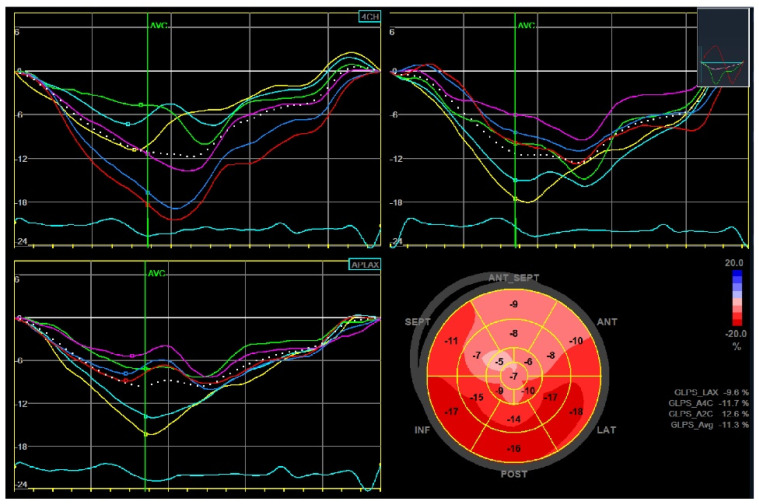
Global longitudinal strain (GLS) of the left ventricle in a patient after anterior ST-segment elevation myocardial infarction. Left ventricular ejection fraction was moderately reduced (40%) and the peak systolic GLS was −11.3%.

**Figure 4:**
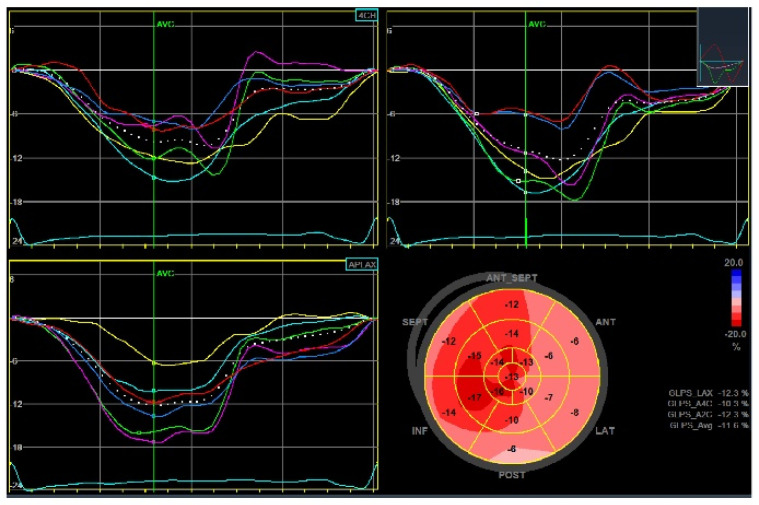
Global longitudinal strain (GLS) of the left ventricle in a patient after non-ST-elevation myocardial infarction revascularized with coronary artery bypass grafting. Left ventricular ejection fraction was preserved (56%) and the peak systolic GLS was −11.6%.

Transthoracic echocardiography is recommended 1–3 months after the index acute event, and periodically in subjects with chronic coronary syndrome, to assess left ventricular function, valvular disease, and hemodynamic status [26]. Emerging data show the role of advanced echocardiography in stratifying these patients beyond the ejection fraction. The left ventricle global longitudinal strain (GLS) is the most widely studied parameter, and its prognostic significance has been evaluated, particularly in patients with CAD. Indeed, peak systolic GLS in patients after ST-elevation MI is an independent predictor of MACE. The cut-off associated with MACE development is <−13% (hazard ratio (HR) between 1.1 and 2.34) [45] (Figure 3). However, other studies have also reported lower GLS values associated with poor prognosis and strong predictors of adverse events (up to > −9.55%) [46,47]. Iwahashi et al. documented the additional role of left ventricular myocardial dispersion in this group of patients for the prediction of MACE with a value > 56.7 ms (HR 1.991, 95% CI 1.033–3.613, *p* = 0.03) [48]. Furthermore, Olsen et al. highlighted a linear association between GLS and MACE and a three-fold increased risk with values > −13.6% in patients after coronary artery bypass grafting [49] (Figure 4). Finally, in patients with chronic coronary syndrome, Espersen et al. showed significant MACE prediction with a low mean GLS value of −14% (HR 1.20, 95% CI 1.00–1.43, *p* = 0.049) [50].

Several issues should be considered when performing an echocardiographic examination. Similar to US exams, this imaging technique is strictly “operator-dependent” and subject to interpretive error. Classical analysis during the echocardiographic examination is the evaluation of regional left ventricle function through the observation of wall thickening and endocardial motion of the single segments, which are assessed based on observer experience and subjective and qualitative considerations. Poor image quality, reduced patient cooperation, artifacts, improperly oriented views, under- or over-estimation of chamber size, and ventricular function are other limitations to consider. Indeed, LVEF could be misleading in the presence of coexisting conditions such as moderate to severe mitral regurgitation, which can lead to an overestimation of left ventricular function [51,52]. Several issues are also involved when using advanced parameters such as Global Longitudinal Strain. This technique can be altered by pitfalls such as low-quality images, left ventricle foreshortening, loading conditions, and software and vendor equipment variability.

**Figure 5:**
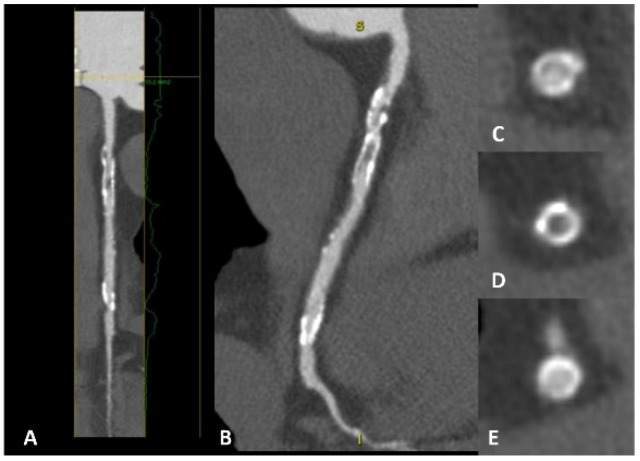
A patient with previous percutaneous revascularization of the right coronary artery, with cardiac CT showing evident in-stent restenosis in both long-axis view (panels **A**,**B**) and short-axis view (panels **C**–**E**). In panel (**D**), a clear hypodensity is evident inside the stent lumen compared with panels (**C**,**E**).

Coronary CT is a well-known non-invasive technique for evaluating patients with CAD. In the context of secondary prevention, this approach provides the initial valuable data required for effectively stratifying these patients (Figure 5). Phenotypic characterization of the plaque aims to define whether a plaque is at high risk. Positive remodeling, spotty calcification, low attenuation plaque, and the Napkin-ring sign are indicators of a high-risk plaque. Furthermore, lesion volume progression is an independent predictor of MACE in subjects with unrevascularized non-culprit intermediate stenosis (50–69%) [53]. In patients with ACS, the characterization of non-culprit plaques is central to risk assessment and management over time. Non-culprit plaques with >50% luminal narrowing or with a high plaque burden are associated with a significant risk of MACE [54]. In addition, coronary CT-derived fractional flow reserve adds prognostic information on non-culprit plaques in patients with ACS. Indeed, a CT-derived FFR value ≤ 0.80 is a predictor of future MACE (HR 1.56, 95% CI 1.01–2.83, *p* = 0.048) [55]. Furthermore, plaque burden progression is associated with a high incidence of MACE in individuals with stable angina [56]. Data are also emerging on the useful role of coronary CT in the risk assessment of individuals with stroke. CAD detection in these patients is independently associated with MACE [57,58], adding prognostic value over the CAC score [59]. Finally, van’t Klooster et al. reported interesting data on the incremental value of CAC, thoracic aortic calcium, and heart valve calcium scores in patients with established CVDs such as coronary heart disease, cerebrovascular disease, and/or PAD [60]. The CAC score improves the performance of risk prediction models and adds prognostic value to the prediction of future MACE in patients with stable CV disease (HR 1.35, 95% CI 1.15–1.58).

CCTA raises concerns, particularly in relation to radiation exposure and the use of iodine contrast agents, that can give rise to significant risks such as hypersensitivity reactions, thyroid dysfunction, and contrast-induced nephropathy [61]. Notably, radiation exposure is associated with potential cancer development [62]. In recent decades, advancements in technology (e.g., step-and-shoot protocols) have enabled a noteworthy decrease in radiation exposure. Moreover, in specific cases, the implementation of high-pitch spiral protocols on modern dual-source machines can lead to a further reduction in ionizing radiation [63,64]. Regarding renal function and contrast agent exposure, recent studies indicate that the contrast media might not directly raise creatinine levels or increase the risk of Acute Kidney Disease (AKI), irrespective of comorbidities that may predispose to nephrotoxicity [65]. This is true both for coronary angiography and CCTA, which work by using iodine contrast agents. Some studies show that CKD alone might not be the problem when performing imaging using iodine contrast, but it might be when there are associated conditions and comorbidities such as hypovolemia. Giving the patients sodium chloride infusions or sodium bicarbonate after performing blood gas analysis might be a solution for preventing contrast-induced AKI. After these considerations, CCTA may be appropriate for subjects with chest pains or anginal equivalent and established coronary artery disease, or for studying the patency of coronary artery bypass grafts or previously implanted coronary stents (preferably for stent diameters ≥ 3.0 mm) [66]. In-stent restenosis (ISR) is a phenomenon in which a previously stented coronary lesion narrows the walls due to myointimal hyperplasia. The frequency of this event has reduced since the introduction of drug-eluting stents over bare metal stents, with the latter being more prone to narrowing. Indeed, the mean time from PCI to ISR is 12 months with drug-eluting stents and 6 months with bare metal stents. This means that, even if reduced, ISR is still an issue, especially in diabetic patients who have the highest risk of ISR. Using CCTA, it is possible to make an early diagnosis of ISR. Some studies show that in almost two-thirds of symptomatic patients with previous coronary stent implantation, ISR can be ruled out using CCTA [66].

CMR shows more consistent findings compared with the other techniques in the risk assessment of patients for secondary prevention. CMR combines heart function and morphology assessment to evaluate ischemic heart disease. This is achieved by analyzing myocardial wall motion and function, as well as evaluating the presence, extent, and characteristics of myocardial edema, ischemia, and scar tissue, similar to echocardiography, but with better reproducibility and less inter-operator variability. CMR can also be used to obtain stress and rest perfusion images whose principles are similar to Myocardial Scintigraphy, but with the benefits of (1) being a first-pass imaging study, hence it is performed using an abbreviated adenosine protocol, and (2) higher spatial resolution (>20×) than radionuclide technique and can detect a perfusion defect that is limited to the subendocardial layer [67]. The third aspect is represented by edema imaging to differentiate between acute and chronic myocardial injuries. Myocardial edema is the result of the activation of the inflammatory cascade, associated with acute ischemic damage, that leads to cell death and activation of the inflammatory response, with accumulation of water and waste products in the injury-related area. These features can be studied with some MRI sequences such as T2-weighted short-tau inversion recovery (STIR), which is a highly T2-weighted sequence that allows enhancement of the presence of fluids at the tissue level without using contrast agents. The fourth feature that CMR can study is represented by infarct imaging in the form of Delayed enhancement (DE) imaging. This phenomenon is associated with the alteration of the cell membranes of the cardiomyocytes in ischemic heart disease, which leads to the accumulation of Gadolinium in infarct-related areas. The diseased myocardium has delayed Gadolinium washout compared with the healthy myocardium, leading to the phenomenon of late gadolinium enhancement (LGE). The extension of gadolinium accumulation is associated with the amount of necrotic tissue. The main issues with CMR are its costs and availability. Even if the availability of CMR in Europe is improving, the cost of MRI is still high for public health, and better training of physicians, especially fellow cardiologists, is needed to improve the radiologist–cardiologist interactions.

**Figure 6:**
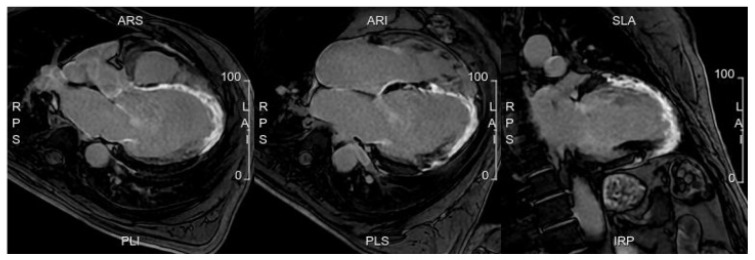
A case from a patient with a recent myocardial infarction. Cardiac MRI evidenced the presence of extensive myocardial left ventricular fibrosis associated with worse prognosis at follow-up.

Several studies have revealed the prognostic value of GLS in patients after ST-segment elevation myocardial infarction (STEMI). A value ≥ −11% is associated with a high MACE rate (HR 1.21, 95% CI 1.11–1.32, *p* < 0.001) [68]. GLS has a higher prognostic value than left ventricular ejection fraction [69]. Moreover, in patients with CAD, the presence of late gadolinium enhancement (LGE) is a strong predictor of MACE, and increasing size is associated with a 4% increase in HR [70] (Figure 6). In STEMI patients, anterior myocardial infarction and a larger extent of damage as assessed by LGE increase the risk of MACE (HR 1.03, 95% CI 1.01–1.06, *p* = 0.01) [71]. In addition, microvascular obstruction (MVO) also predicts MACE in these patients in the long term [72], and late MVO extent > 0.385 g is a strong independent predictor [73]. Another useful parameter for risk stratification in patients with reperfused STEMI is the presence of intramyocardial hemorrhage (IMH) as assessed using T2 imaging. Indeed, IMH is associated with an increased risk of MACE regardless of the left ventricular ejection fraction [74,75]. During the acute phase, infarct and global extracellular volume (ECV) predict MACE with hazard ratios of 4.04 and 5.10, respectively [76]. Finally, prognostic data on the study of non-infarct-related coronary artery territory are also emerging in patients with STEMI. Indeed, T1 > 1250 ms in non-infarcted myocardium correlates with an increased risk of MACE (HR 2.534, 95% CI 1.033–6.219; *p* = 0.042) [77]. The presence of MVO in these territories is also associated with worse cardiovascular outcomes [78].

## 4. New Perspectives for Secondary Prevention

Residual risk assessments of patients for secondary prevention are based on clinical and laboratory variables. Currently, further stratification of these patients using cardiovascular imaging through risk calculators is lacking. Patients with ASCVD or poly-vascular disease are not in a single category with the same risk. Indeed, individualized risk assessment is needed to guide therapy and intensify treatment in patients with high residual risk.

Studies of risk prediction models suggest that prognostic imaging parameters are necessary. Risk calculators for residual risk assessment should include clinical, laboratory, and imaging parameters. Currently, the SMART risk score is used for assessing the risk of recurrent events for secondary prevention. Future research should investigate the potential of adding cardiovascular imaging parameters to this score. Finally, the current literature on cardiovascular imaging for secondary prevention is mostly based on individuals who have suffered from AMI. New data are needed for patients who have suffered a stroke, aortic aneurysm, or PAD. In addition, there is a paucity of data on subjects with poly-vascular atherosclerotic disease, and, for this population that is at very high risk, an accurate assessment is necessary to stratify risk.

The possibility of assessing effective cardiovascular risk in patients with poly-vascular atherosclerotic disease status using imaging techniques may be improved by correlating imaging data with metabolic targets or with different medical therapies to verify their effectiveness in preventing the progression of CVDs.

RCTs of patients with different metabolic targets of serum LDL cholesterol and Hb1Ac levels, as well as different anti-diabetic therapies in terms of CAC scores, would be useful for comparing the benefits of medical therapies, ideal targets for specific patients, and the progression of CVDs in differently stratified patients.

## 5. Gaps in Evidence and Future Implications

### 5.1. Calcium Scoring and Beyond for Primary Prevention

Calcium scoring was developed in the 1980s by Agatston but has only recently gained popularity in the primary prevention guidelines, particularly the use of statins [18]. New automated image segmentation techniques are now used routinely to quantify coronary calcium with high precision and reproducibility. New CAC-based strategies for restratifying individual risks reclassify as many as 20% of patients compared with prior guidelines [79]. Calcified plaque is a direct sign of CAD, whereas previous guidelines employed a probabilistic approach based on risk factors for CAD. A CAC score = 0 and CAC > 1000 have also been established as low-risk and high-risk entities, respectively, but the intermediate values remain debatable, especially in populations of younger individuals, women, and patients with diabetes. New methods for measuring CAC-based risk have been proposed, including refinement of the total calcium Agatston method. In particular, it is important to account for the number of plaques (diffusivity index), the volume of plaques, localization with up-risking of proximal plaque, and calcium density profiles, as the combination of some, if not all, of these elements has been reported to improve risk assessment in asymptomatic individuals [80,81]. Furthermore, non-calcified atherosclerosis as seen on CCTA may partly explain the residual risk seen in patients with low CAC scores, as the calcified component represents only one-fifth of the total plaque burden.

### 5.2. Advances in CAD Assessment Using CCTA

Coronary plaque detection and stenosis quantification are now well-developed. The use of coronary CT has recently been standardized to the CAD–RADS score [82]. However, non-invasive characterization of plaque vulnerability remains a challenge in all modalities. Coronary angiography-based approaches using intravascular ultrasound (IVUS) or optical coherence tomography (OCT) have been proposed but are inherently limited by invasiveness and cost. Recently, the new CCTA guidelines have included new CAD prognosis-related parameters, including disease extension (P parameter) and plaque vulnerability (V parameter) criteria such as the presence of positive arterial wall remodeling, hypodense lipid core, microcalcifications, and the presence of hyperdense peripheral napkin ring, which is a more meaningful sign of plaque complication. While the guidelines are commendable for extending the CCTA information from diagnosis to prognostic parameters, we should note that the aforementioned parameters are still visually assessed and are thus susceptible to reader subjectivity. Current CCTA is hampered in this regard by its spatial resolution of 0.5–0.6 mm, but a new technology based on photon counting now achieves 0.2 mm isotropic spatial resolution, which significantly enhances the ability to image atherosclerotic plaque components. Importantly, this is achievable with even lower doses of contrast agents and radiation. New AI-based algorithms and post-processing methods will greatly benefit from increased image quality and enable robust automated plaque quantification.

Interest in the use of artificial intelligence in medicine, especially imaging exams, has grown over the last few years. The main applications of AI in cardiovascular imaging include CCTA detection of stenosis, post-processing of the obtained images using CMR, and automatized segmentation of the heart chambers during LGE.

In CCTA, AI can be a useful tool for efficient and rapid evaluation of atherosclerotic plaques. Recent studies have shown that AI evaluation of the plaques, to ground truth of consensus of the readers, has a high level of accuracy, sensitivity, specificity, positive predictive value (PPV), and negative predictive value (NPV) in detecting coronary stenosis [83]. Furthermore, another potential application of AI in CCTA is the study of the functional features of stenosis using the fractional flow reserve Computed Tomography (FFRct) tool. FFR derived from CCTA has an excellent correlation with invasive FFR and remains diagnostically robust in the presence of reduced signal-to-noise ratio (SNR), coronary calcification, and motion artifacts [84].

In MRI, AI applications mostly involve post-processing data such as segmentation and tissue characterization. The main characteristic of automatic segmentation is represented by the possibility of obtaining results, such as manual segmentation, in a short time, allowing faster evaluation of the obtained images, which otherwise would require long post-processing using manual or semi-automated software [85]. Furthermore, the latest CCTA developments enable native spectral imaging, which can combine iodine imaging for high contrast resolution angiography and greater soft tissue and calcium resolution from multiple energy spectra to better assess plaque components. Taken together, these technological advances in both hardware and software will hopefully yield an enhanced assessment of plaque vulnerability features.

Going beyond coronary wall analysis, the assessment of peri-coronary fat using CCTA is possible and has been validated using a transcriptomic approach to define a fat attenuation index (FAI) associated with coronary inflammation and a new risk biomarker [86]. A simpler and more global approach that measures total epicardial fat in 3D using CCTA (or non-contrast CCT) has also linked volume and mean densities of epicardial adipose tissue with atherosclerotic burden and risk [87]. Automated AI-based segmentation methods for total and peri-coronary epicardial adipose tissue have also been validated and will help in the clinical application of this approach after population-specific relevance and position have been defined relative to other tests [88].

The main perspective in individual risk assessment remains the definition of multiparametric risk scores, including several imaging modalities as well as clinical and biological data. As an illustration, we showed that total and epicardial adipose tissue, measured using CCTA in combination with blood IL6 levels, are the best predictors of short-term mortality in patients with diabetes and COVID-19, opening an avenue to target patients for preventive corticosteroid therapy that avoids a cytokine storm and subsequent complications [89,90].

### 5.3. Advances in MRI for Risk Assessment

One of the most striking advances in MRI is the ability to characterize myocardial fibrosis non-invasively, hence detecting arrhythmogenic and/or heart failure without the need for endomyocardial biopsy. This will help in restratifying individual risks, for example in hypertrophic cardiomyopathy (HCM), where the presence of delayed enhancement >15% of LV mass has been associated with adverse outcomes [91]. The advent of quantitative measurements of scar tissue and diffuse fibrosis via extracellular volume (ECV) is promising for a better definition of individual prognosis of several conditions, e.g., dilated cardiomyopathy, sarcoidosis, amyloidosis, and myocarditis [92]. New biomarkers such as the intracellular and extracellular mass components of LV mass measured using CMR T1 mapping have been proposed for monitoring the effect of hormones such as aldosterone or cortisol on the myocardium in vivo [93,94]. Such biomarkers may be promising new therapeutic targets.

Another innovative application of MRI is the measurement of cardiovascular age by assessing the ascending aortic distensibility (AAD). This imaging biomarker is the earliest and most specific marker of cardiovascular aging in humans. It has been associated with MACE and mortality independently of all established risk factors in a large general population [95,96]. We have shown that AAD is associated with premature CAD (before the age of 45 years) and with increased stiffness, a marker of accelerated aging in diabetes, hypertension, obesity, and Marfan syndrome or bicuspid aortopathy. Importantly, AAD may prove to be an important new index of aortic aneurysm severity and vulnerability in ongoing studies. Currently, half of aortic dissections occur below the recommended threshold for prophylactic surgery. This new functional index of aortic wall properties combined with flow-derived indices from 4D-flow may prove beneficial for better assessment of aortic risk and guide surgical decisions.

## 6. Conclusions

The role of cardiovascular imaging is underrated in primary as well as secondary prevention, even though it can improve the recognition of cardiovascular risk in patients. Future trials should determine the role of CV imaging, its therapeutic implications for primary prevention, and how the identification of poly-vascular diseases may involve the intensification of medical strategies for determining different metabolic targets in this high-risk population.
